# UC-MSCs promote frozen-thawed ovaries angiogenesis via activation of the Wnt/β-catenin pathway in vitro ovarian culture system

**DOI:** 10.1186/s13287-022-02989-8

**Published:** 2022-07-15

**Authors:** Wenjuan Xu, Caiyun Wu, Xiaoqian Zhu, Jingjing Wu, Zhiguo Zhang, Zhaolian Wei, Yunxia Cao, Ping Zhou, Jianye Wang

**Affiliations:** 1 Reproductive Medicine Center, Department of Obstetrics and Gynecology, The First Affiliated Hospital of Anhui Medical University, No 218 Jixi Road, Hefei, 230022 Anhui China; 2 Anhui Province Key Laboratory of Reproductive Health and Genetics, Anhui Medical University, No 81 Meishan Road, Hefei, 230032 Anhui China; 3 NHC Key Laboratory of Study On Abnormal Gametes and Reproductive Tract (Anhui Medical University), No 81 Meishan Road, Hefei, 230032 Anhui China

**Keywords:** Umbilical cord mesenchymal stem cells, Frozen-thawed ovaries, In vitro ovarian culture system, Angiogenesis, Wnt/β-catenin pathway

## Abstract

**Background:**

Ovarian tissue cryopreservation and transplantation are novel therapeutic approaches for fertility preservation. However, follicle loss caused by ischemic and hypoxic damage is one of the issues after frozen-thawed ovarian tissue transplantation. Promoting angiogenesis in grafts is the key to restore cryopreserved ovarian function. Mesenchymal stem cells (MSCs) have been reported to facilitate angiogenesis in the cryopreserved ovarian tissue transplantation. However, the risk of embolization, immunogenic effect and tumorigenesis hinders the clinical application of MSCs to human organ transplantation. In this study, we established an in vitro ovarian culture system to restore frozen-thawed ovarian function before transplantation with the application of umbilical cord mesenchymal stem cells (UC-MSCs), and explored the effects of UC-MSCs on frozen-thawed ovaries in vitro ovarian culture system and the mechanisms of UC-MSCs on the angiogenesis of frozen-thawed ovaries.

**Methods:**

A simple in vitro three dimensional (3D) ovarian culture system using Matrigel was established to support to an ideal niche, and ovary was alone cultured in the 24-well plate as a control. We also evaluated the effects of UC-MSCs treatment on ovarian function with or without Matrigel support. All thawed ovaries were randomly divided into control group (Matrigel−/UC-MSCs−), Matrigel group (Matrigel+/UC-MSCs−), UC-MSCs group (Matrigel−/UC-MSCs+) and UC-MSCs + Matrigel group (Matrigel+/UC-MSCs+). HE staining was used to detect the histological structure of follicles and TUNEL staining was used to detect cell apoptosis. The number of microvessels was counted to evaluate neovascularization. The mRNA expression of VEGFA, IGF1 and ANGPT2 were detected by RT-PCR. Western blotting was used to measure the expression of GSK-3β, β-catenin and p-β-catenin.

**Results:**

In the absence of UC-MSCs, 3D culture system supported by Matrigel showed significantly improved follicular development and microvascular number. Additionally, UC-MSCs were also found to effectively improve follicular development and microvascular number regardless of the culture condition used. However, alleviated follicular apoptosis, increased mRNA expression of angiogenesis-related gene and activated Wnt/β-catenin pathway occurred only in the UC-MSCs + Matrigel group. Besides, with the application of IWP-2 in UC-MSCs + Matrigel group, Wnt//β-catenin pathway could be blocked by IWP-2 serving as one of Wnt/β-catenin pathway inhibitors.

**Conclusions:**

This in vitro study showed the beneficial effects of UC-MSCs on thawed ovaries and explored a potential mechanism inducing angiogenesis. In particular, 3D ovarian culture system supported by Matrigel further improved UC-MSCs treatment. The in vitro culture system using Matrigel and UC-MSCs may provide a potential treatment strategy for improving the success rate of thawed ovaries transplantation.

**Supplementary Information:**

The online version contains supplementary material available at 10.1186/s13287-022-02989-8.

## Background

Ovarian tissue cryopreservation has been considered as a promising strategy to restore endocrine function and fertility for young cancer patients who suffer from chemotherapy and/or radiotherapy [1, 2]. To date, more than 130 live births have been reported after transplantation of frozen-thawed ovarian tissues worldwide [3]. However,

the initial revascularization takes 3–5 days after human ovarian tissue xenotransplantation, and frozen-thawed ovarian tissues are exposed to ischemic and hypoxic damage in the early post-transplantation phase [4], and inevitable ischemia plays a major role in follicular loss due to failure of angiogenesis after transplantation [5]. Further research has demonstrated enhancing neovascularization can shorten the ischemic period [6, 7]. Thus, how to promote angiogenesis in grafts is the key to restore cryopreserved ovarian function.

Mesenchymal stem cells (MSCs) are widely used in regenerative medicine because of their easy isolation from multiple tissues, self-renewal potential and differentiation into various cell types [8]. The angiogenic effects of MSCs on damaged ovarian function have been proved [9–11]. In addition, human umbilical cord mesenchymal stem cells (UC-MSCs) on a collagen scaffold transplantation have an obvious effect on regulating angiogenesis [12]. MSCs can upregulate vascular endothelial growth factor (VEGF), fibroblast growth factor 2 (FGF2) and angiopoietin (ANGPT) expression, improve neovascularization, and increase blood perfusion in the cryopreserved ovarian tissue transplantation [13]. Although these outcomes are encouraging, most of MSCs transplanted into injured tissues will exhaust due to lacking oxygen and nutrients [14]. The effectiveness and long-term safety of transplanting MSCs into patients need to be evaluated. And the risk of embolization, immunogenic effect and tumorigenesis hinders the clinical application of MSCs to organ transplantation [15–17].

Therefore, we establish an in vitro ovarian culture system to apply MSCs to thawed ovaries before transplantation, which can improve ovarian function and avoid the potentially unfavorable impacts of MSCs transplantation. Three dimensional (3D) organ culture system is considered as a promising and suitable system for in vitro ovarian culture system, which better mimic physiological structures than the two dimensional (2D) culture system [18, 19]. According to the previous investigations, in vitro 3D ovarian culture system using Matrigel and activin A supplementation favors cavity formation of follicles and has higher maturation and fertilization rates of oocytes [20]. In this study, we used Matrigel to establish an in vitro 3D ovarian culture system, and evaluated the effects of UC-MSCs treatment on ovarian function with or without Matrigel support. It has been unknown that whether UC-MSCs could recover ovarian function by promoting angiogenesis in vitro 3D ovarian culture system, and whether 3D ovarian culture system using Matrigel could improve UC-MSCs treatment. Here we describe a simple in vitro culture system to explore the effects of UC-MSCs on the frozen-thawed ovaries.

## Materials and methods

### Ovary collection, cryopreservation and thawing

Eight-week-old BALB/c female mice were provided by Boyuan Experimental Products Department (Hefei, China). The mice were weighed and executed by cervical dislocation to obtain the ovaries. All animal experiments were approved by the Institutional Animal Care and Use Committee of Anhui Medical University. After washing the tissues with phosphate-buffered saline (PBS, Gibco, USA) twice at room temperature, ovary cryopreservation was performed using Medium 199 (Sigma, USA) supplemented with 100 IU/ml penicillin and streptomycin (Hyclone, USA) for 10 min [21]. And ovaries were transferred to 10 ml of 5% dimethyl sulfoxide (DMSO, Sigma, USA) and 5% ethylene glycol (EG, Sigma, USA) and 10 ml of 10% DMSO and 10% EG in Medium 199 for 10 min respectively [22, 23]. Each ovary was directly put into an open vessel filled with liquid nitrogen, and the vitrified ovarian tissue was put into the precooled marked freezing tube. After tightening the lid, the freezing tube was put into liquid nitrogen for storage. Ovaries were thawed 7–10 days later. Freezing tubes were first immersed in a 37 °C water bath with gentle shaking. And ovaries were transferred in turn to the following three thawing solutions for 5 min respectively: (i) 10 ml of 0.5 M sucrose in Medium 199, (ii) 10 ml of 0.25 M sucrose in Medium 199, and (iii) 10 ml Medium 199 [24, 25].

### In vitro ovarian culture system

**Fig. 1:**
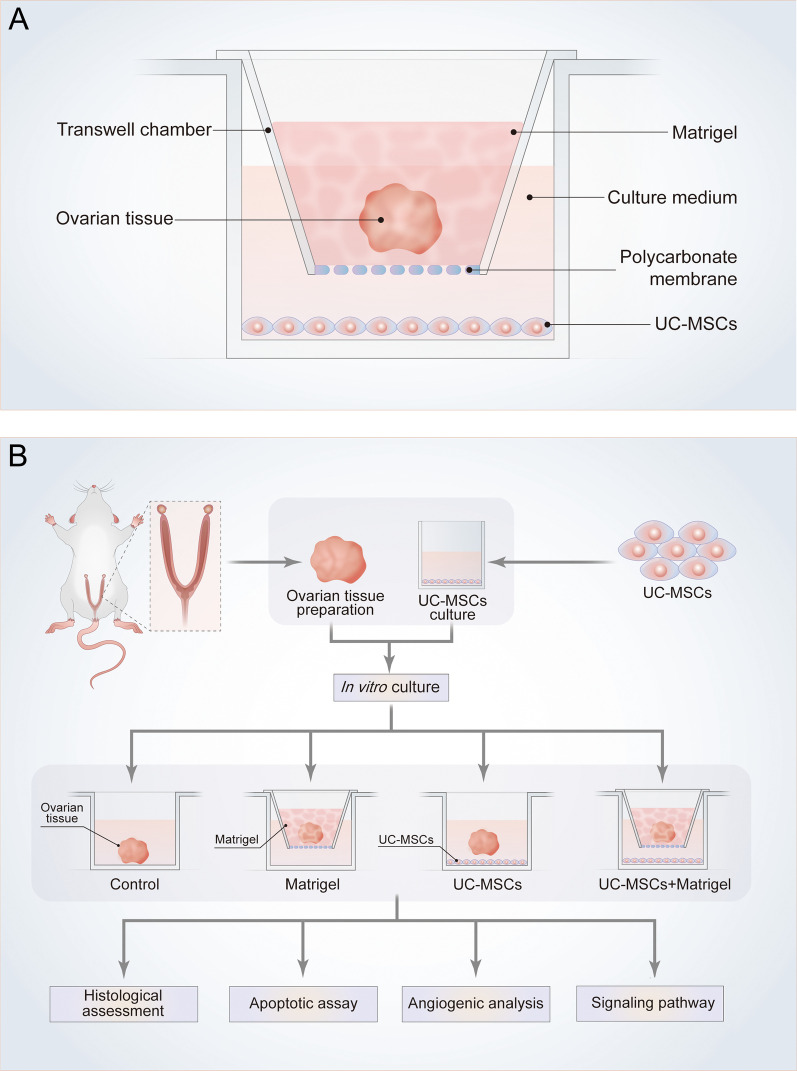
Schematic description of the experimental design. **a** Thawed ovary encapsulated with Matrigel was placed in a transwell chamber. The transwell chamber was inserted into a 24-well plate pre-seeded with UC-MSCs. **b** The schematic and detailed procedure used to explore the effects and mechanisms of UC-MSCs on thawed ovaries in vitro culture system

UC-MSCs were isolated from the connective tissue of the human umbilical cord as described [26]. After enzymatic digestion, UC-MSCs were cultured in expansion medium consisting of Dulbecco’s modified Eagle’s medium (DMEM)/F-12 supplemented with 10% (v/v) fetal bovine serum (FBS, Gibco, USA). UC-MSCs were planted in the 24-well plate one day in advance and cultured in DMEM/F-12 medium supplemented with 10% FBS, 100 IU/ml penicillin, and 100 µg/ml streptomycin. After washing UC-MSCs with PBS twice, the transwell chamber (0.4 µm, Polycarbonate, Corning, USA) was put into the 24-well plate (Corning, USA). The cryopreserved ovary encapsulated with Matrigel (BD Biocoat, Corning, USA) was placed in the upper chamber. Matrigel was diluted with DMEM/F-12 medium (1:1) and 150 µl were placed on polycarbonate membrane to mimic ovarian microenvironment. Because of the permeability of polycarbonate membrane, the substances secreted by UC-MSCs could affect the ovary in the upper chamber while whole ovary would not migrate down into the lower chamber. The lower chamber was added with 800 µl of DMEM/F-12 medium. In order to evaluate the effects of UC-MSCs treatment on the structure and function of thawed ovaries cultured with or without Matrigel support, all thawed ovaries were randomly divided into four groups: (i) control group, thawed ovary alone without Matrigel or UC-MSCs; (ii) Matrigel group, thawed ovary encapsulated with Matrigel was placed in the upper chamber; (iii) UC-MSCs group, co-culture of thawed ovary and UC-MSCs without Matrigel; (iiii) UC-MSCs + Matrigel group, thawed ovary encapsulated with Matrigel was placed in the upper chamber while UC-MSCs were planted in the lower chamber. Figure 1a and b respectively show the diagram of in vitro ovarian culture system and the technical route of this study for a better understanding of the entire experiment.

### Histologic staining and follicle counting

After 4 days of in vitro ovarian culture system, the ovaries were recovered for histological evaluation, fixed in 4% formaldehyde and embedded in paraffin. Serial 5 μm paraffin sections were cut for the following hematoxylin and eosin (H&E) staining to evaluate follicle growth. Five discontinuous sections were stained with H&E and observed under an epifluorescence microscope with the ZEN image program. All follicles in whole ovary were counted in a 200-fold field. According to the standard described previously [27], primordial follicles were defined as an oocyte surrounded by a single layer of flat granular cells; primary follicles were classified with an oocyte surrounded by a single layer of cubic granular cells; secondary follicles were cubic granular cells having two or more layers with no visible antral cavity; antral follicle were classified with an antral cavity with follicular fluid; and the corpus luteum consisted of lutein cells.

### Immunofluorescence and immunohistochemistry

To detect apoptosis of ovaries, the cultured ovaries were recovered for TUNEL staining. According to the instruction manual, the terminal deoxynucleotidyl transferase-mediated dUTP nick end labeling (TUNEL) assay was performed using the TUNEL Staining Kit (Elabscience, China). The enzyme TdT (deoxyribonucleic acid terminal transferase) attaches labeled dUTP to the exposed 3'-OH ends of the broken DNA, and labels apoptotic cells by adding fluorescent dUTP to these ends, allowing detection by fluorescence microscopy. Green represents apoptotic signals. Blue represents DAPI-stained nuclei. For each ovary, five fields at 400-fold fields were selected randomly under confocal laser scanning microscope and the average of apoptotic intensity value was calculated. To evaluate neovascularization of ovarian tissue, the ovaries were recovered for immunostaining with anti-CD31 antibodies (Cell Signaling, USA). CD31, as a marker of newly formed vessels, usually exists in vascular endothelial cells and brown coloring of blood vessels represents positive staining. For each ovary, positively stained vessels were identified and quantified at 200-fold field under microscope. The results were calculated as the number of microvessels per ovary.

#### RT-PCR

Total RNA of ovary was extracted by Trizol Reagent (Ambion, Carlsbad, CA, USA), and cDNA was synthesized using a cDNA Synthesis Kit (Takara bio, Japan). PCR amplification was carried out with specific primers (Table 1) under the following conditions: 37 °C for 15 min, 85 °C for 5 s and end at 4 °C. β-actin (ACTB) was used as an internal control gene for normalization [28]. The level of VEGFA [29], insulin-like growth factor 1 (IGF1) [30] and angiopoietin 2 (ANGPT2) [31] mRNA expression was calculated by the following formula: 2^−ΔΔCt^. The Ct of VEGFA, IGF1 and ANGPT2 was compared with that of the reference ACTB gene.

**Table 1:** Primers used for qPCR validation

Gene	Primer sense	Primer antisense
ACTB	CTACCTCATGAAGATCCTGACC	CACAGCTTCTCTTTGATGTCAC
VEGFA	GCCAGGGACGGAGAAGGAGTC	GCAGAACCACAGAGCGACAGC
IGF1	GTGAGCCAAAGACACACCCA	ACCTCTGATTTTCCGAGTTGC
ANGPT2	AGCTAACCTCTTTCTGCAAAGA	GCTTCCTTTCTTTCCACAGATG

### Western blotting

Ovaries were collected and homogenized in lysis buffer and protease inhibitor cocktai containing 1 mM dithiothreitol (DTT), 1 mM phenylmethylsulfonyl fluoride (PMSF), 1 mg/ml leupeptin, 2 mg/ml aprotinin, and 5 mM ethyl glycol tetraacetic acid (EGTA, Beyotime Biotechnology, China). Then the tissues were centrifuged at 12,000× *g* for 10 min to obtain supernatants which were diluted to 1 µg/µl with 4 × sample buffer and frozen at − 20 °C. Proteins were quantified with the Lowry Assay. The tissue extracts were resolved on 10% sodium dodecyl sulfate–polyacrylamide gel electrophoresis (SDS-PAGE) and transferred to polyvinylidene fluoride (PVDF) membranes. The membrane was blocked and incubated with primary antibodies of anti-β-actin (1:1000, Cell Signaling, USA), anti-β-catenin (1:1000, Cell Signaling, USA), anti-p-β-catenin (1:1000, Cell Signaling, USA) and anti-GSK-3β (1:1000, Cell Signaling, USA) at 4 °C overnight, then reacted with a secondary antibodies of anti-rabbit IgG (1:1000, Cell Signaling, USA) after removing unconjugated antibodies by tris-buffered saline Tween-20 (TBST). The peroxidase activity was visualized using an enhanced chemiluminescence (ECL) kit (Biosharp, USA) to image the proteins recognized by the antibodies.

### Statistics analysis

All experiments were repeated at least three times. The data are presented as the mean ± standard deviation (SD) and analyzed with SPSS 26.0 software (IBM, Armonk, NY, USA). ANOVA was used for comparison among four groups and LSD was used for the post-hoc test. A value of *P* < 0.05 indicates statistically significant.

## Results

### UC-MSCs improved recovery of thawed ovaries

**Fig. 2:**
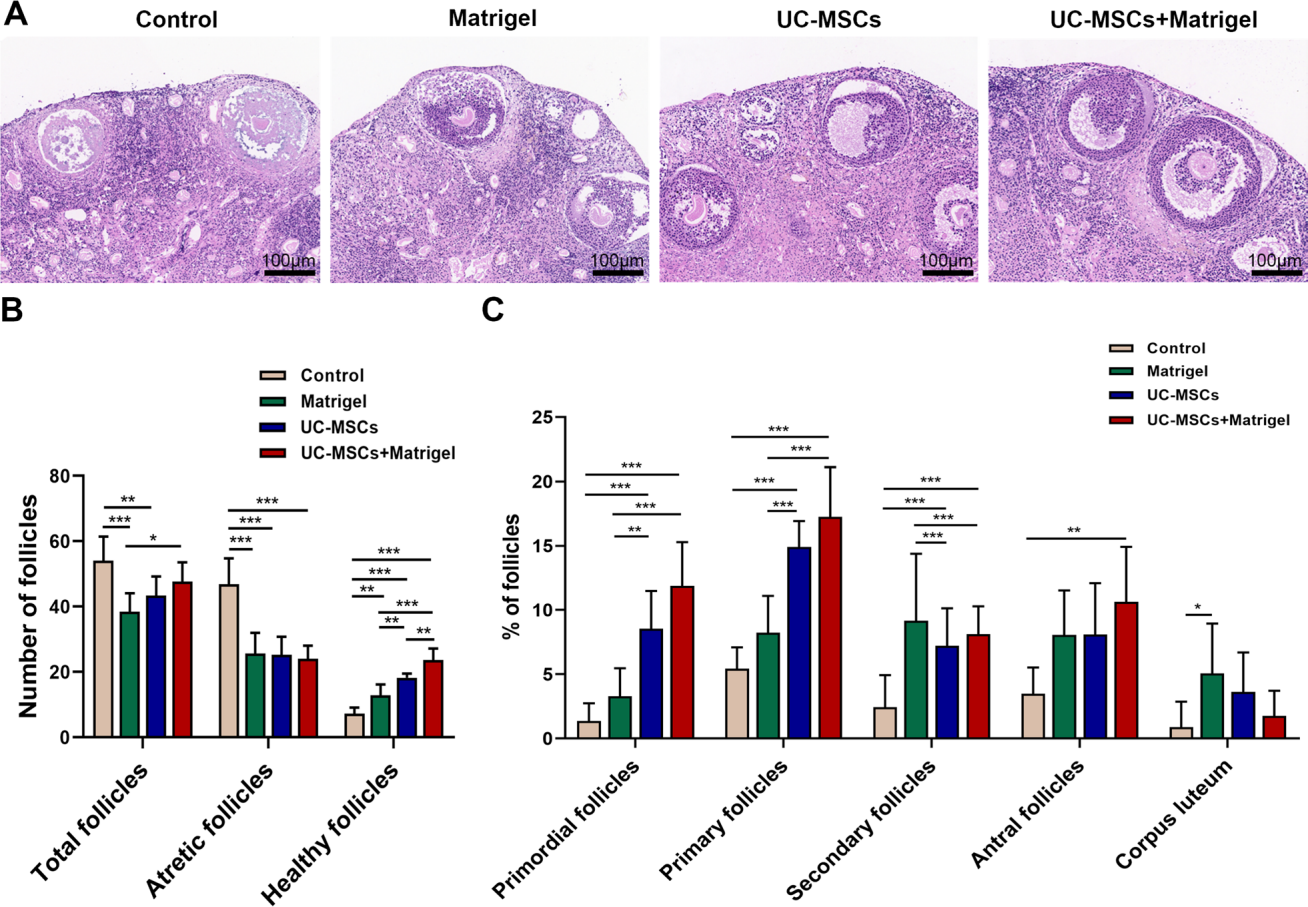
UC-MSCs improved the recovery of thawed ovaries in vitro culture system. **a** Ovarian histology was analyzed after 4-day in vitro culture using H&E staining. Scale bar = 100 µm. **b** The number of follicles in the four groups. **c** The percentage of follicles at different stages in the four groups. Bar represents mean ± SD, n = 5 (biological replicates); *P < 0.05, **P < 0.01 and ***P < 0.001

Ovaries were observed histologically by H&E staining at a high magnification (Fig. 2a) and follicles in different developmental stages were classified and counted. Compared with the control group (Matrigel−/UC-MSCs−), the UC-MSCs + Matrigel group showed similar total follicle number. Nevertheless, the number of atretic follicles was significantly reduced under the treatment of UC-MSCs and Matrigel and the number of healthy follicles was significantly increased (Fig. 2b). It is notable that in the absence of UC-MSCs, 3D culture system supported by Matrigel also showed significantly improved follicular development (Fig. 2b). In addition, the proportion of primordial and primary follicles in UC-MSCs and UC-MSCs + Matrigel groups were higher than those in control and Matrigel groups (Fig. 2c, *P* < 0.05). Similarly, UC-MSCs and UC-MSCs + Matrigel groups showed greatly increased percentage of secondary follicles compared to control group (Matrigel−/UC-MSCs−) (Fig. 2c, *P* < 0.05). Histological analysis revealed that the administration of UC-MSCs promoted relatively normal folliculogenesis in in vitro ovarian culture system with or without Matrigel.

### UC-MSCs suppressed apoptosis of thawed ovaries in vitro 3D ovarian culture system

**Fig. 3:**
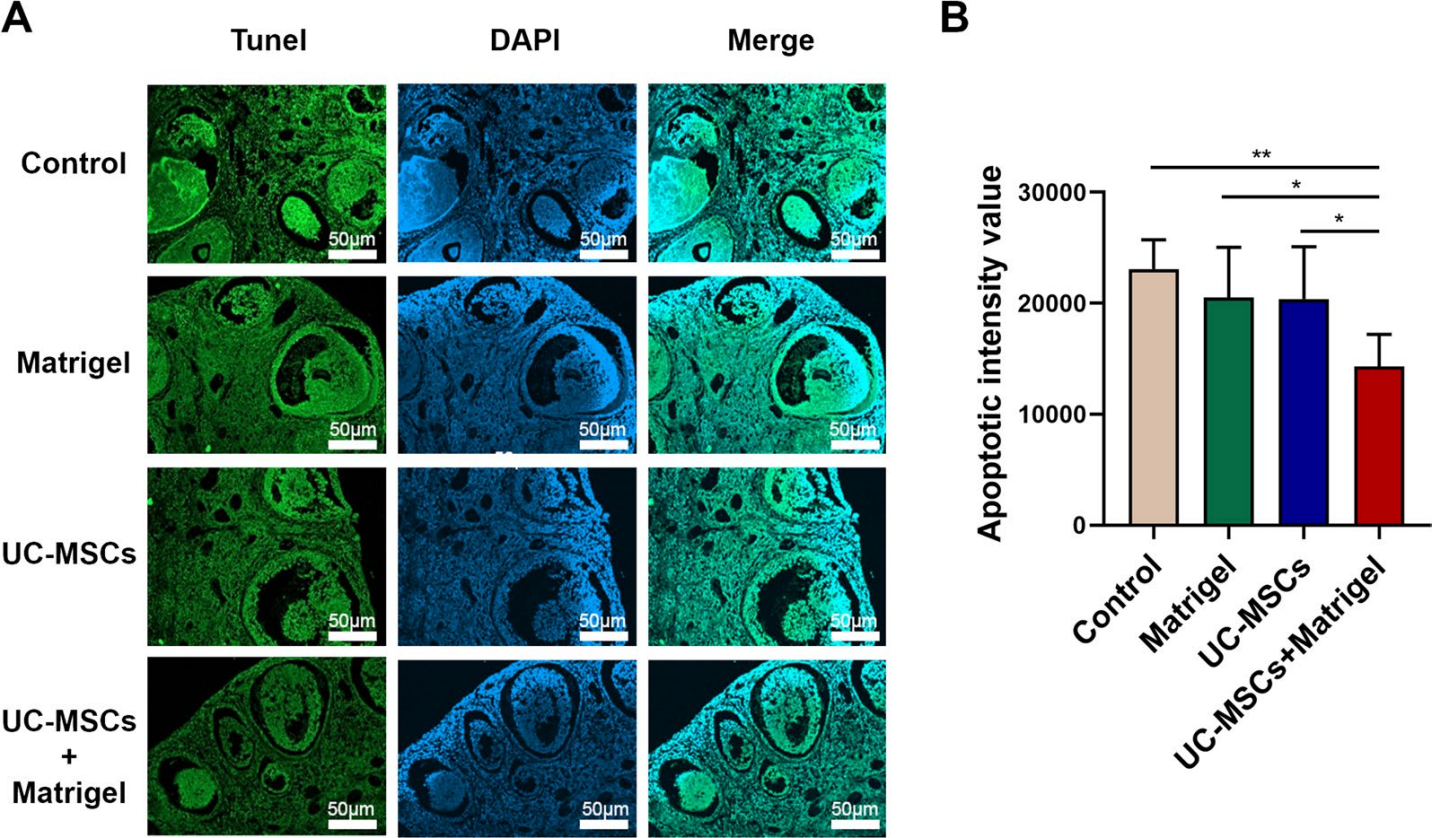
UC-MSCs decreased the apoptosis of thawed ovaries in vitro 3D culture system. **a** Representative images of cell apoptosis in the four groups after immunofluorescence staining. Green represents apoptotic signals. Blue represents DAPI-stained nuclei. Scale bar = 50 µm. **b** Fluorescence intensity was quantified. Bar represents mean ± SD, n = 5 (biological replicates); *P < 0.05 and **P < 0.01

TUNEL staining was used to determine follicular apoptosis in the thawed ovaries. After TUNEL staining, ovary sections were observed under confocal laser scanning microscope (Fig. 3a), and the apoptotic intensity mean value was quantified. Compared with the control (Matrigel−/UC-MSCs−, Additional file 1: Fig. S1), Matrigel and UC-MSCs groups, the apoptotic intensity mean value was significantly decreased in UC-MSCs + Matrigel group (Fig. 3b, *P* < 0.05). These results indicated that UC-MSCs could suppress the apoptosis of thawed ovaries only in vitro 3D ovarian culture system.

### UC-MSCs promoted angiogenesis of thawed ovaries in vitro 3D ovarian culture system

**Fig. 4:**
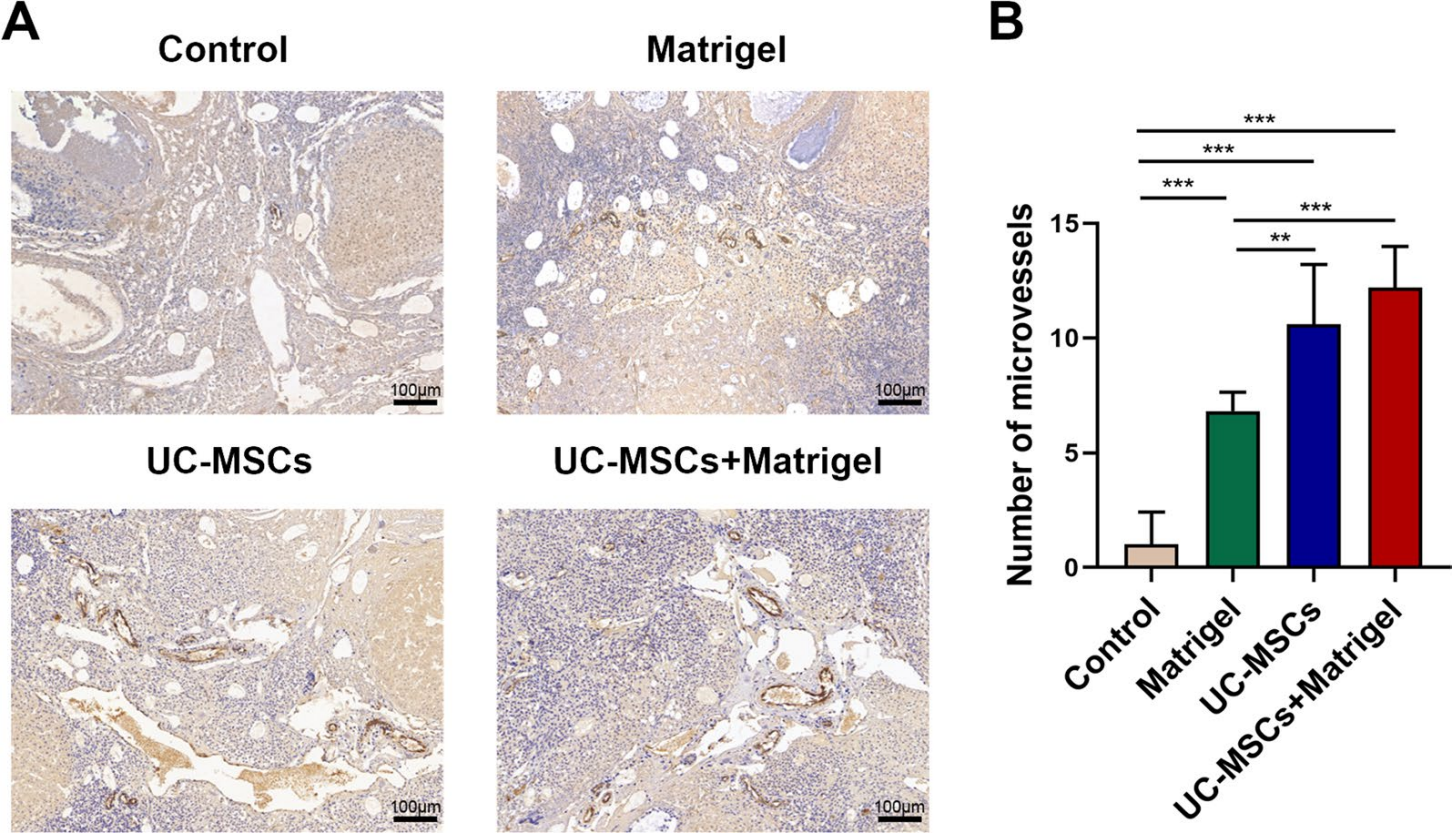
UC-MSCs increased the microvessels of thawed ovaries in vitro culture system. **a** Representative images of ovarian microvessels in the four groups using CD31 immunohistological staining. Brown represents positive staining vessels. Scale bar = 100 µm. **b** The microvascular density was quantified. Bar represents mean ± SD, n = 5 (biological replicates); **P < 0.01 and ***P < 0.001

**Fig. 5:**
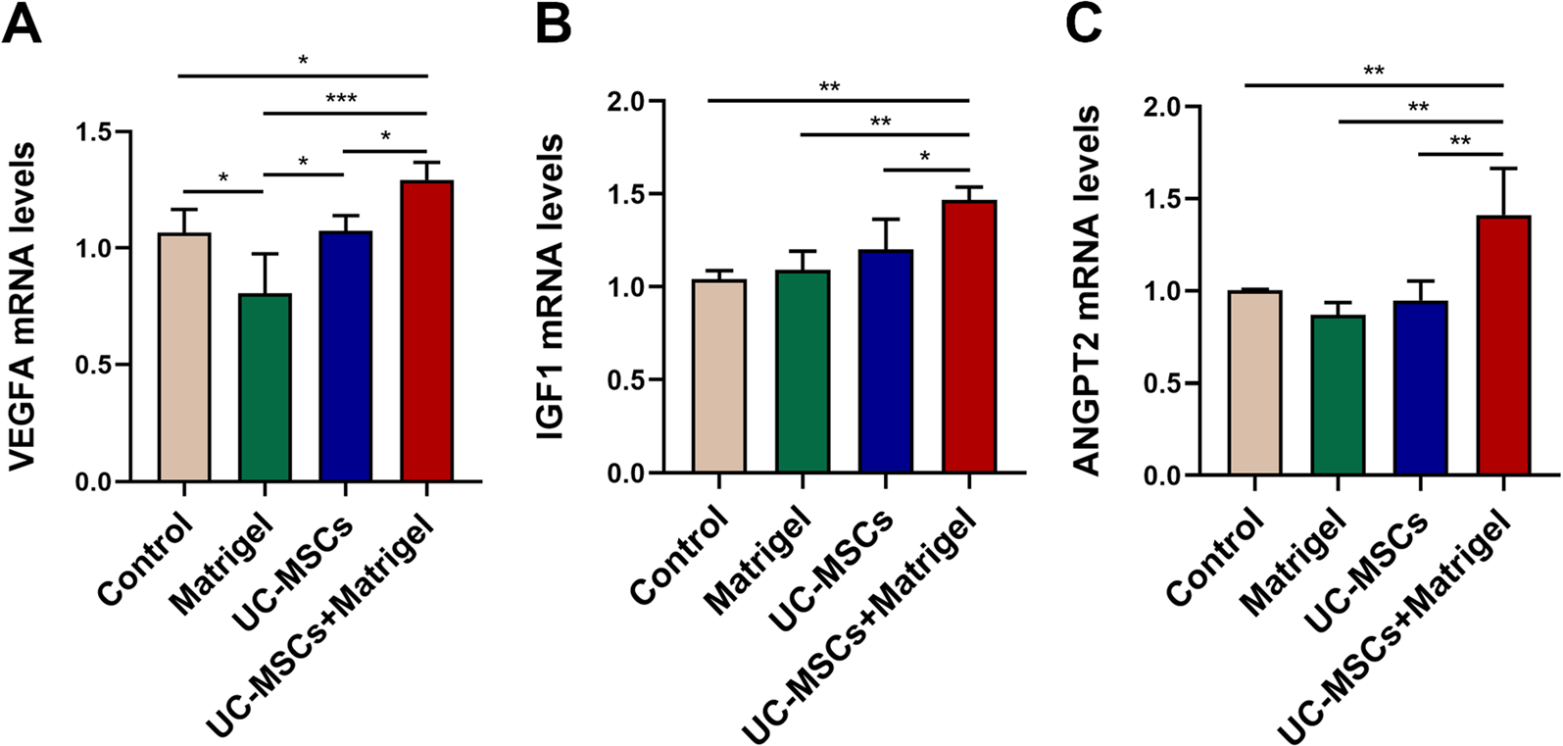
UC-MSCs improved the expression of angiogenesis-related growth factors of thawed ovaries in vitro 3D culture system. The mRNA expression of VEGFA (**a**), IGF1 (**b**), and ANGPT2 (**c**) in the four groups. Bar represents mean ± SD, n = 3 (biological replicates); *P < 0.05, **P < 0.01 and ***P < 0.001

To examine the angiogenesis of thawed ovaries, the number of microvessels was counted via CD31 staining. As was shown in Fig. 4a, the number of microvessels in control group (Matrigel−/UC-MSCs−) was apparently less than that in other experimental groups (*P* < 0.05). Although UC-MSCs + Matrigel group showed relatively more neovascularization than UC-MSCs group, no statistical difference was observed between two groups (Fig. 4b). Furthermore, RT-PCR analysis showed that the mRNA expression levels of angiogenesis-related gene VEGFA, IGF1 and ANGPT2 in the UC-MSCs + Matrigel group were significantly higher than those in the other groups (Fig. 5a–c, *P* < 0.05). These results suggested that UC-MSCs increased the endogenous angiogenesis of thawed ovaries in vitro 3D ovarian culture system.

### UC-MSCs activated the Wnt/β-catenin pathway to enhance vascularization

**Fig. 6:**
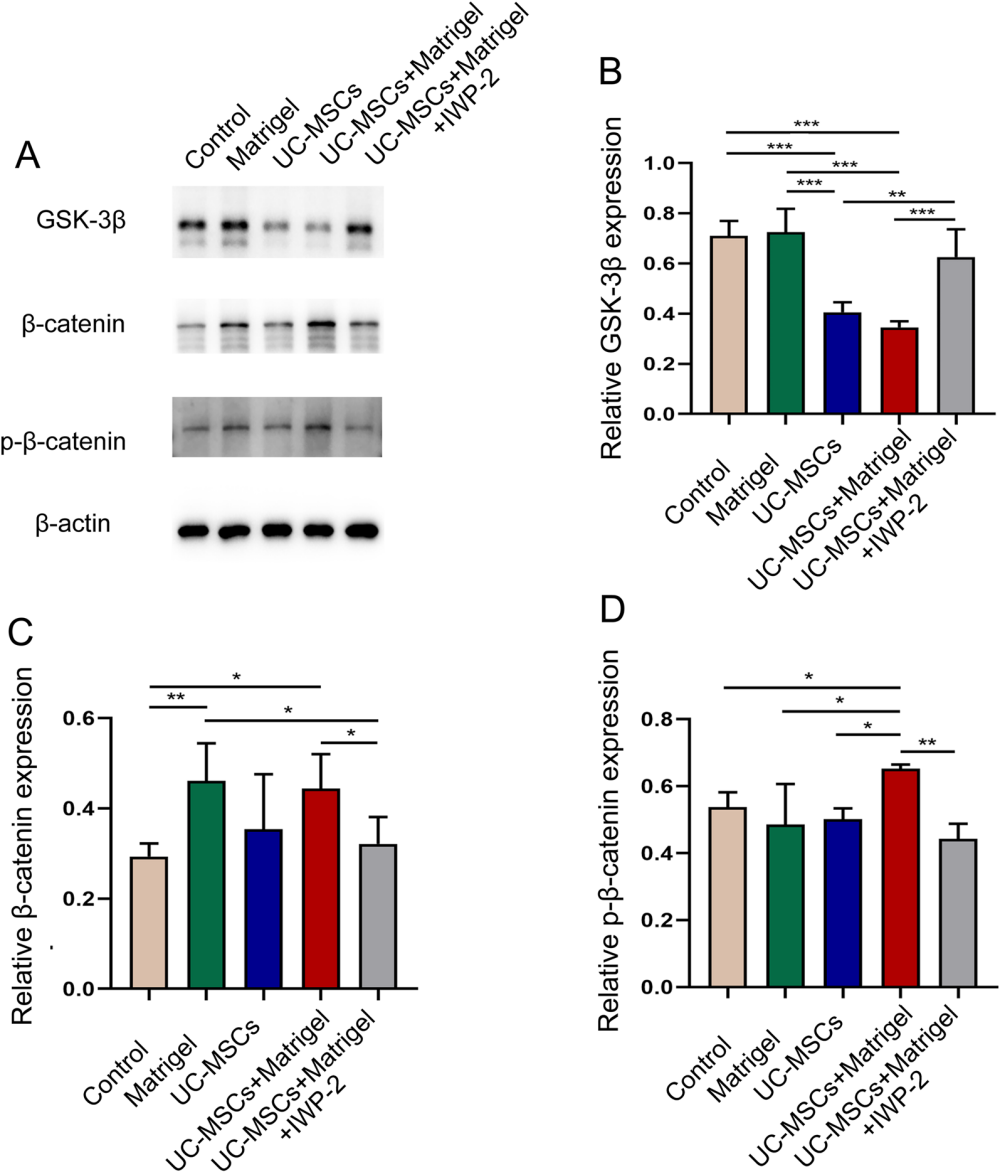
UC-MSCs activated Wnt/β-catenin signaling pathway in vitro 3D ovarian culture system. Representative images of protein expression in the five groups using western blot (**a**). The protein expression of GSK-3β (**b**), β-catenin (**c**) and p-β-catenin (**d**) in the five groups. Bar represents mean ± SD, n = 3 (biological replicates); *P < 0.05, **P < 0.01 and ***P < 0.001

The Wnt/β-catenin signaling pathway plays an important role in angiogenesis. β-catenin is a key regulator in the Wnt signaling pathway. Normally, β-catenin remains low expression in cells and the Wnt signaling pathway is turned off. When the Wnt pathway is activated, the function of GSK-3β is inhibited, and β-catenin is separated from the APC/AXIN/GSK3β complex and accumulated in the cytoplasm, and then enters the nucleus to replace the transcriptional repressor Groucho and bind to the transcription factor TCF/LEF, thereby activating the transcription of many target genes and promoting cell proliferation and differentiation [32]. The degradation of GSK-3β and the accumulation of β-catenin in cytoplasm are known as markers of Wnt/β-catenin signaling pathway activation. Phosphorylation of β-catenin at Ser552 (p-β-catenin) also induces β-catenin accumulation in the nucleus and increases its transcriptional activity [33]. To further investigate the mechanisms of UC-MSCs on vascularization in vitro culture system, the expression of GSK-3β, β-catenin and p-β-catenin were measured. As was shown in Fig. 6, the expression of GSK-3β in the ovaries of UC-MSCs + Matrigel group was significantly downregulated, whereas the expression levels of β-catenin and p-β-catenin were upregulated compared with the other three groups. In addition, IWP-2 as a Wnt/β-catenin pathway inhibitor was used in UC-MSCs + Matrigel group, the GSK-3β expression was significantly upregulated and the β-catenin and p-β-catenin expression were downregulated (Fig. 6b–d, *P* < 0.05). The western blot results confirmed that UC-MSCs could activate the Wnt/β-catenin pathway to improve the vascularization of thawed ovaries in vitro 3D culture system.

## Discussion

Ovarian tissue cryopreservation and transplantation is beneficial to restore endocrine function and fertility for prepubertal girls and young women who suffer with cancer [1, 2]. However, these techniques are limited by early ischemia and hypoxia after the cryopreserved ovarian tissue transplantation [34]. Ovarian neovascularization can shorten the ischemic period and overcome ischemia and hypoxia [7, 35]. In this study, we investigated the effects of UC-MSCs on thawed ovaries recovery in an in vitro co-cultured model, and our results indicated that UC-MSCs exerted the therapeutic effects on the structure and function of thawed ovaries in vitro culture system. In particular, 3D ovarian culture system supported by Matrigel further improved UC-MSCs treatment.

UC-MSCs play a critical role in tissue regeneration, repair and vascularization [36, 37], and UC-MSCs exhibit higher proliferative potential and multidirectional differentiation [38]. Due to ease of isolation, painless collection, abundant distribution and low immunogenicity [39], UC-MSCs are considered as an ideal resource of stem cells in our study. Our results showed that, regardless of Matrigel used or not, administration of UC-MSCs in vitro could promote follicular survival, including a decrease in the proportion of atretic follicles and an increase in that of normal follicles. And the proportion of normal follicles at different stages of development except corpus luteum were significantly increased, in particular primordial and primary follicles. Previous findings have demonstrated that insufficient vascular supply restricts follicular growth and causes atresia [40]. In addition, studies have proved that MSCs induce angiogenesis in ischemia animal models [41], which is consistent with our results. The number of microvessels in the ovaries co-cultured with UC-MSCs was significantly increased, which suggested that UC-MSCs were effective in angiogenesis of thawed ovaries. And this improvement may contribute to follicle survival. As well as in vivo, UC-MSCs were found to effectively improved follicular development and microvascular number in vitro with or without Matrigel support. In the absence of UC-MSCs, 3D culture system supported by Matrigel also showed significantly improved follicular development and microvascular number.

Numerous efforts have been devoted to techniques for culturing ovarian tissue or isolated follicles or oocytes in vitro, and these culture techniques can be used to investigate both follicle recruitment, assembly and hormonal requirements of follicular development in addition to acquisition of steroidogenic capabilities during follicular growth [20, 42, 43]. However, the culture of isolated follicles, oocytes or ovarian tissue pieces fails to simulate different interactions among follicles and other cell types in the intact structures [44, 45]. Therefore, in vitro whole ovary culture was utilized to research the morphological or functional changes of ovary in this study, and it could be easily controlled and adjusted. Moreover, 3D organ culture has been demonstrated to better sustain crosstalk between different cell types and maintained the physiological architecture and function [19]. As an ideal hydrogel scaffold, Matrigel has been used extensively for 3D culture system [46]. In our study, we used Matrigel to construct an in vitro 3D ovarian culture system. It is interesting to note that only UC-MSCs + Matrigel group exhibited a significant decrease in follicular apoptosis, while UC-MSCs group was similar to the control (Matrigel−/UC-MSCs−) and Matrigel group. In addition, an obvious increase in VEGFA, IGF1 and ANGPT2 mRNA expression was observed only in the UC-MSCs + Matrigel group. These results appear to contradict experimental evidence from others that UC-MSCs therapy alleviates ovarian injury and improves ovarian function, including inhibit apoptosis and increase the expression of VEGFA, IGF1 and ANGPT [47, 48]. This phenomenon may be ascribed to the possibility that UC-MSCs are unable to act as well in vitro as in vivo, further confirming 3D organ culture could mimic the in vivo microenvironment. Therefore, UC-MSCs could suppress ovarian apoptosis and enhance the expression level of angiogenic factors in vitro 3D culture system.

Angiogenesis is essential to provide normal follicular development, ovulation and corpus luteum formation with necessary nutrients, oxygen and hormonal support [49, 50]. ANGPT is essential for regulating angiogenesis, and the targeted inhibition of ANGPT via short-hairpin RNAi (shANGPT) or anti-ANGPT monoclonal neutralizing antibodies (ANGPT Ab) could notably suppress the MSC-stimulated angiogenesis [51]. In addition, MSCs could promote the formation of new bloods partly through a paracrine mechanism by secreting VEGF, IGF1 and other angiogenic growth factors [52]. MSCs could also upregulate the expression of VEGF and VEGF receptor 2 and activate VEGF pathway through the PI3K/AKT/mTOR pathway and the Wnt/β-catenin pathway stimulation [10]. It is notable that Wnt/β-catenin signaling pathway plays an essential role in vascular development under normal and pathological conditions [53]. Several studies have shown that Wnt/β-catenin signaling pathway can regulate the proliferation of endothelial cells while FrzA/sFRP-1 as an antagonist of Wnt protein can inhibit the proliferation of endothelial cells [54, 55]. Wnt/β-catenin signaling pathway can regulate the expression level of angiogenic factors, such as VEGFA, VEGFC and bFGF, which may be the downstream targets of Wnt/β-catenin signaling pathway [56], and this is consistent with our results about RT-PCR. In this study, we also found the expression level of GSK-3β in the UC-MSCs + Matrigel group was greatly lower than that in other three groups, while the expression level of β-catenin and p-β-catenin were upregulated. As a Wnt/β-catenin pathway inhibitor, IWP-2 was used in UC-MSCs + Matrigel group, the GSK-3β expression was significantly upregulated and the β-catenin and p-β-catenin expression were downregulated, suggesting UC-MSCs could activate the Wnt/β-catenin signaling pathway and enhance neovascularization in in vitro 3D ovarian culture system. In the future, we will transplant ovaries after in vitro 3D culture into castrated mice to further evaluate the endocrine function and fertility.

## Conclusion

In conclusion, we demonstrated that UC-MSCs could promote angiogenesis of the thawed ovaries via activation of the Wnt/β-catenin signaling pathway, improve follicular development and alleviate follicular apoptosis in vitro 3D ovarian culture system. This simple in vitro culture system using Matrigel and UC-MSCs may provide a theoretical basis and clinical potential for the further application in patients with preserving fertility.

## Supplementary Information

**Additional file 1. Figure S1.** Representative images of cell apoptosis in the negative controls of immunofluorescence staining. Green represents apoptotic signals. Blue represents DAPI-stained nuclei. Scale bar = 50μm, n=5 (biological replicates).

## Data Availability

All data generated or analyzed during this study are included in this published article.

## Abbreviations

MSCs: Mesenchymal stem cells
UC-MSCs: Human umbilical cord mesenchymal stem cells
VEGFA: Vascular endothelial growth factor A
FGF2: Fibroblast growth factor 2
DMSO: Dimethyl sulfoxide
EG: Ethylene glycol
TUNEL: Terminal deoxynucleotidyl transferase-mediated dUTP nick end labeling
CD31: Cluster of differentiation 31
AKT: Serine/threonine kinase
IGF1: Insulin-like growth factors 1
ANGPT2: Angiopoietin 2
PI3K: Phosphoinositide 3-kinase
mTOR: Mammalian target of rapamycin
bFGF: Basic fibroblast growth factor
GSK-3β: Glycogen synthase kinase-3
