# Supplementary material for: UC-MSCs promote frozen-thawed ovaries angiogenesis via activation of the Wnt/β-catenin pathway in vitro ovarian culture system
Source: Stem Cell Res Ther. 2022 Jul 15;13:296. doi: 10.1186/s13287-022-02989-8 (PMC9284710; doi:10.1186/s13287-022-02989-8)
Supplement: Supplementary file 1 — Additional file 1. Figure S1. Representative images of cell apoptosis in the negative controls of immunofluorescence staining. Green represents apoptotic signals. Blue represents DAPI-stained nuclei. Scale bar = 50μm, n=5 (biological replicates). [file 13287_2022_2989_MOESM1_ESM.docx]

Supplementary Material


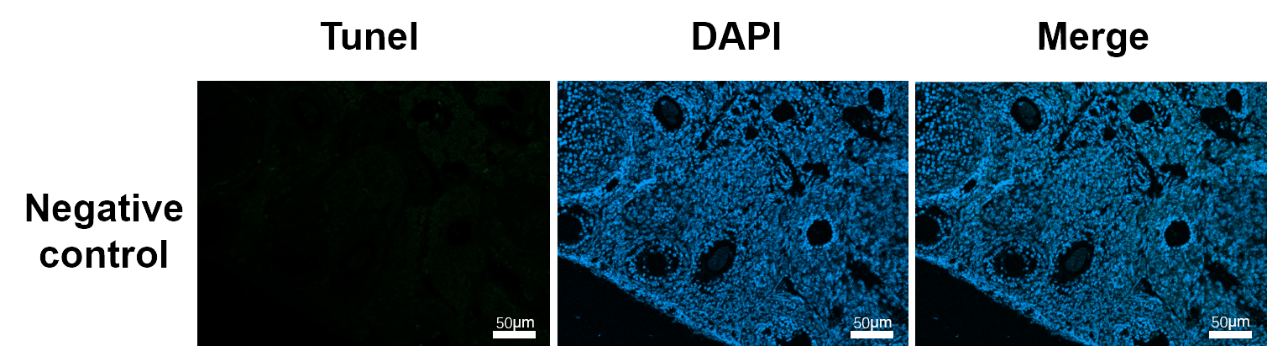


Figure S1. Representative images of cell apoptosis in the negative controls of immunofluorescence staining. Green represents apoptotic signals. Blue represents DAPI-stained nuclei. Scale bar = 50μm, n=5 (biological replicates).
